# Acute Formation of a Blunt Trauma-induced Vertebral Artery Arteriovenous Fistula Treated with Endovascular Occlusion of Vertebral Artery

**DOI:** 10.7759/cureus.5801

**Published:** 2019-09-29

**Authors:** Michael Young, Ryan Johnson, Ajeet Gordhan

**Affiliations:** 1 Neurosurgery, Advocate Bromenn Medical Center, Normal, USA; 2 Neurointerventional Radiology, OSF Healthcare, Bloomington, USA

**Keywords:** blunt traumatic injury, arteriovenous fistula, endovascular occlusion, vertebral artery

## Abstract

Fistulous cerebrovascular injuries can occur spontaneously, iatrogenically following surgical procedures, or can result as a consequence of penetrating trauma. To our knowledge, this is only the second reported case of blunt-trauma induced cervical vertebral artery arteriovenous fistula (AVF) formation in a 55-year-old male. This was successfully occluded with N-butyl cyanoacrylate (NBCA) embolization of the recipient vein and endovascular coil ligation of the vertebral artery.

## Introduction

Arteriovenous fistulas (AVF) of the vertebral artery are rare vascular malformations of the spinal vasculature that can present after cervical spine surgery, central venous catheterization, chiropractic manipulations, diagnostic cerebral angiography, percutaneous nerve blocks, radiation therapy, penetrating traumatic injury, or even more rarely blunt traumatic injury. We present a case of a 55-year-old male who sustained an AVF of the vertebral artery after a blunt traumatic mechanism. This vertebral artery AVF was treated promptly with an endovascular sacrifice of the parent vertebral artery with subsequent obliteration of this fistula. 

## Case presentation

**Figure 1:**
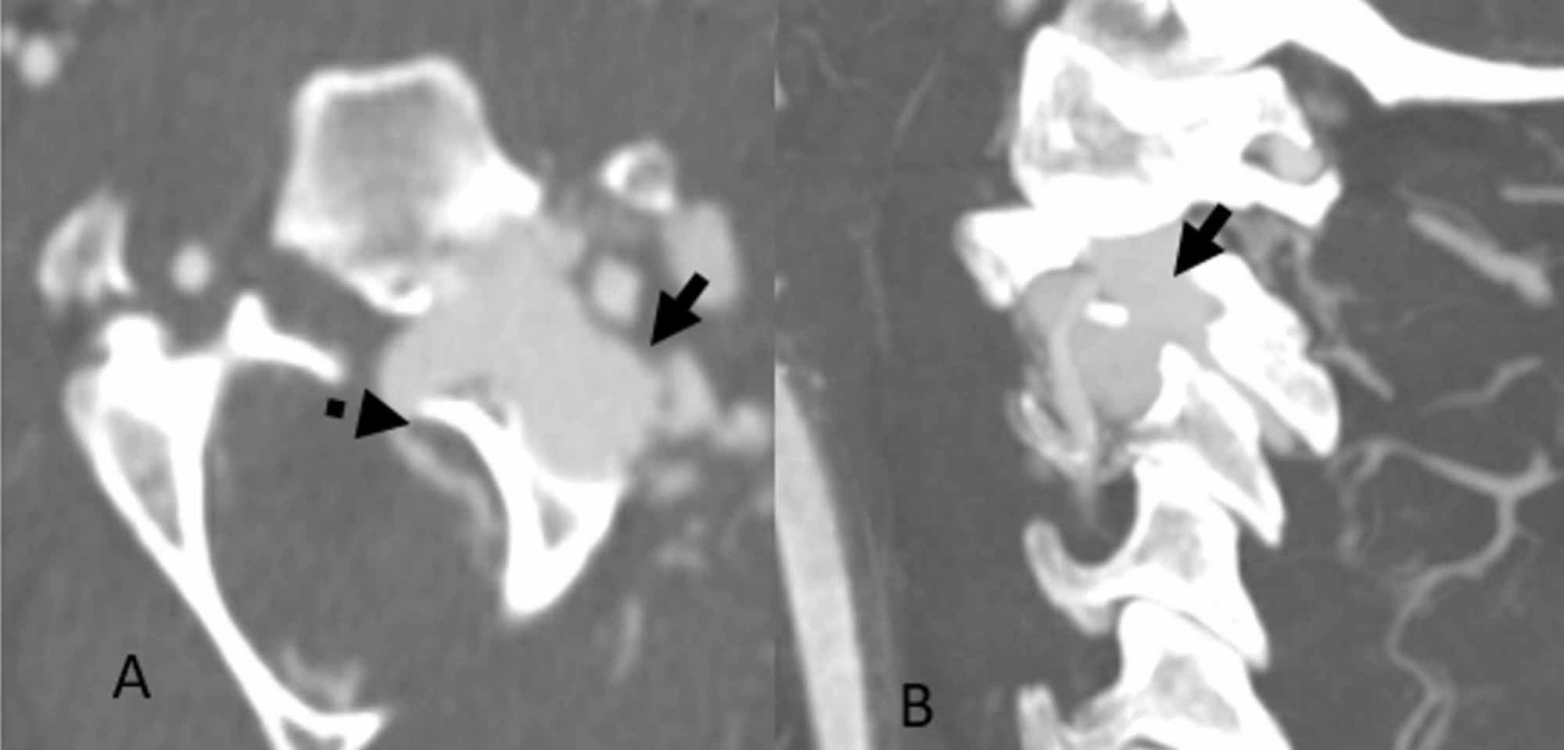
A: Fracture through the left transverse foramen at C3 (dotted arrow); B: Extravascular contrast accumulation within the left transverse foramen, contiguous with dominant left vertebral artery (solid arrow)

A 55-year-old male with past medical history significant for schizophrenia presented to the emergency department (ED) after being found unresponsive outside by family members at his residence. The presumed mechanism was a traumatic fall off the patient’s porch, but this was unwitnessed. Paramedics reported a Glasgow coma score (GCS) of 3 in the field and he was intubated for airway protection. He was hemodynamically stable on arrival to the ED. Neurologically, his pupils were equal and reactive to light, and brainstem reflexes were all intact. He did not display any movements to noxious stimuli in all his extremities. Computed tomography (CT) and computed tomography angiography (CTA) of the head and cervical spine were significant for unstable vertical shear fractures involving the posterior cortices of C1, C2, C3, and C4. Additionally, there was evidence of contrast extravasation from the distal V2/proximal V3 segment of the left vertebral artery (Figure 1A, 1B). To further evaluate for left vertebral artery injury, the patient underwent a catheter-based digital subtraction angiogram (DSA) on the first day of hospitalization.

**Figure 2:**
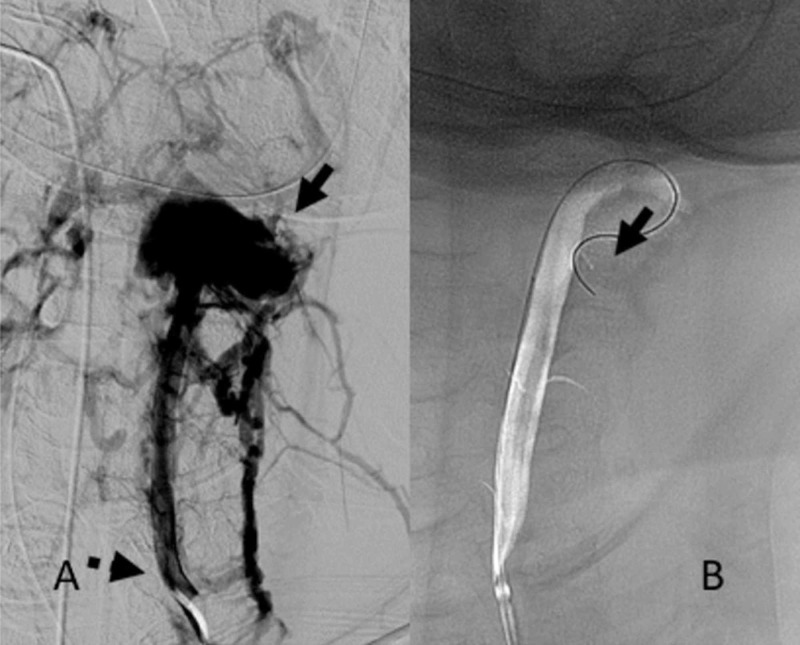
A: Left vertebral artery digital subtraction angiogram (dotted arrow), demonstrating extravasation of contrast within an ectatic venous recipient (solid arrow) through a high flow arteriovenous fistulous connection and opacification of the surrounding draining veins; B: Microcatheter navigation into the venous recipient for endovascular embolization with N-butyl cyanoacrylate

**Figure 3:**
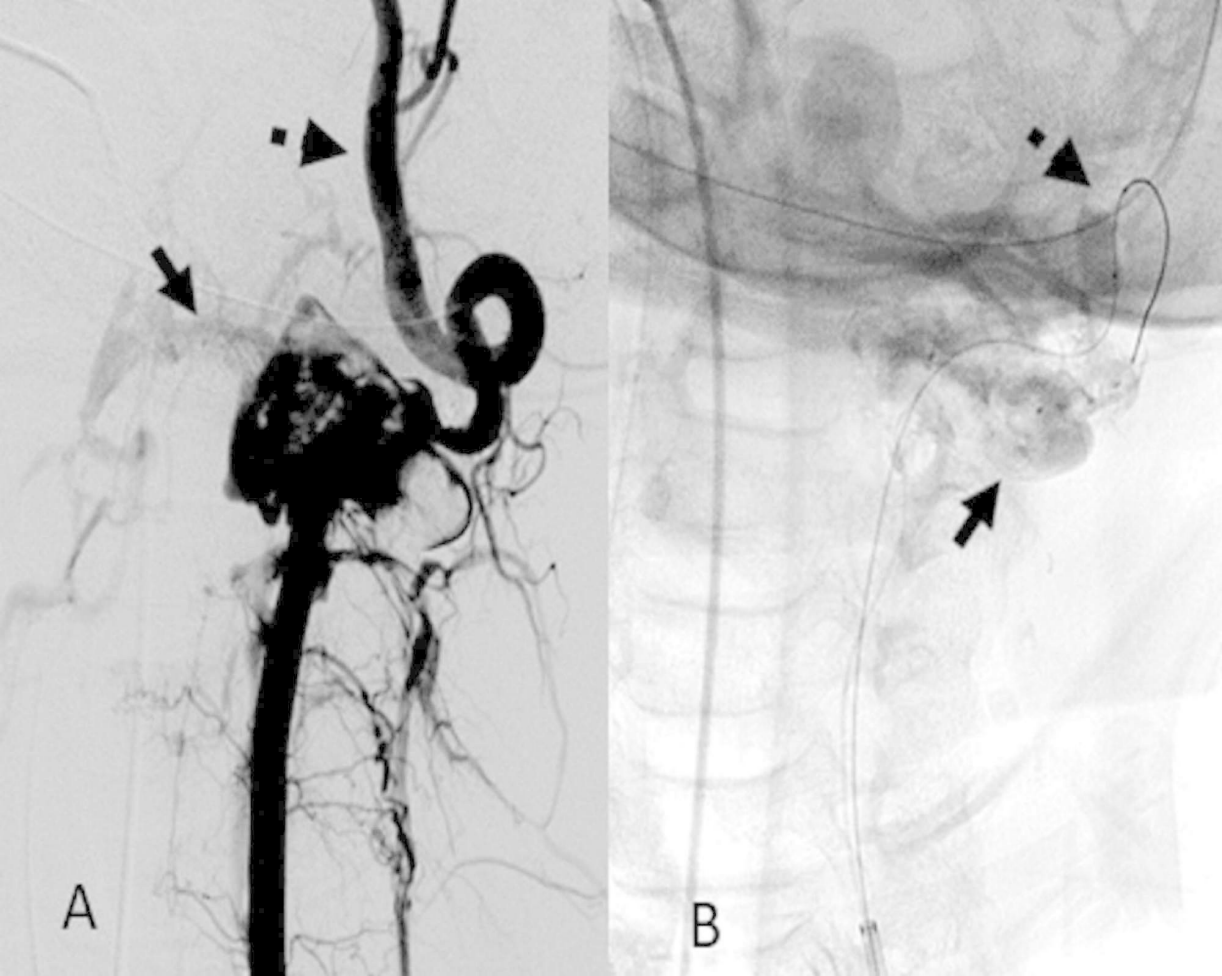
A: Reduced flow within fistula with opacification of the vertebral artery distal to the fistula and persistent opacification of the paraspinal veins (solid arrow); B: N-butyl cyanoacrylate cast in venous recipient (solid arrow) and inflated balloon (dotted arrow) within vertebral artery distal to fistula

**Figure 4:**
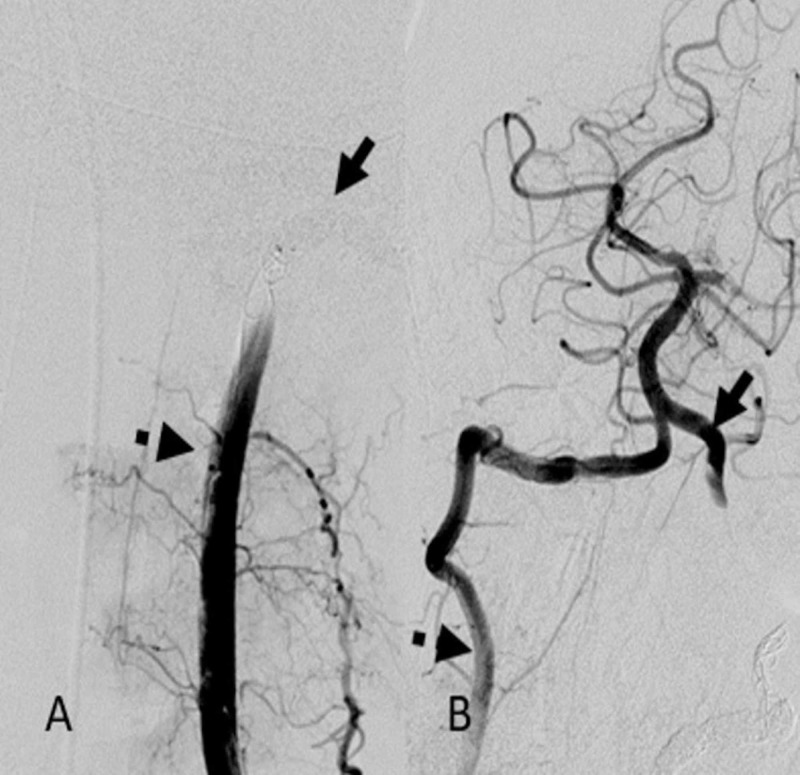
A: Coil artifact in distal vertebral artery (solid arrow), proximal to fistula with complete disconnection of the shunt and stasis in the left vertebral artery (dotted arrow); B: Right vertebral artery DSA (dotted arrow) with reflux into distal left vertebral artery and the left posterior inferior cerebellar artery DSA, digital subtraction angiogram

DSA demonstrated an AVF at the left V2/V3 vertebral artery junction with an enlarged ectatic recipient vein draining into the perimedullary and deep venous structures (Figure 2A). In anticipation of surgical stabilization of the cervical spine fractures, endovascular disconnection of the shunt vascularity was performed under general anesthesia. An Excelsior SL-10 (Stryker, Kalamazoo, MI, USA) microcatheter was used to catheterize the recipient vein. N-butyl cyanoacrylate (NBCA) of 3 mL was injected under fluoroscopic observation (Figure 2B). This resulted in a reduction of fistulous flow through the AVF (Figure 3A). A 4 mm x 20-mm TransForm Balloon catheter (Stryker, Kalamazoo, MI, USA) was then advanced through the distal aspect of the left cervical vertebral artery to prevent reflux of the embolic material into the basilar artery. An Excelsior SL-10 microcatheter was then advanced into the residual AVF, and an additional 3 mL of NBCA was injected (Figure 3B). The TransForm Balloon catheter was removed, and subsequent angiographic imaging revealed persistent filling of the AVF. Due to the pending cervical spine surgery, the decision at this time was to endovascularly ligate the left vertebral artery to avoid stent placement and the need for dual antiplatelet therapy. Ligation of the left vertebral artery was accomplished with the deployment of nine Target 360 detachable coils (Stryker, Kalamazoo, MI, USA), and two Tornado embolization coils (Cook Medical, Bloomington, IN, USA; Figure 4A). This resulted in complete angiographic obliteration of the left vertebral artery with adequate flow through the non-dominant right vertebral artery, basilar artery and the left posterior inferior cerebellar artery (Figure 4B). 

On post-procedure day one, the patient improved neurologically, demonstrated by an improvement in GCS from 3T to 8T. He was localizing to noxious stimuli with the left upper extremity and withdrawing to noxious stimuli in the left lower extremity. He would open his eyes to voice. On post-procedure day two, the patient underwent a posterior occipito-cervical stabilization with occipital titanium cables, lateral mass screws, and connecting rods for his unstable vertical shear fractures from C2 to C4. There were no intraoperative or perioperative complications and the patient was transferred to the intensive care unit postoperatively.

His neurologic status slowly improved throughout the remainder of his hospitalization. Prior to discharge, he was able to open his eyes spontaneously and track objects across his visual fields bilaterally. His brainstem reflexes remained intact. He did not follow commands but continued to localize in the left upper extremity. His right upper and lower extremities withdrew to noxious stimuli. His GCS was 10T upon discharge.

In order to better prognosticate rehabilitation potential, an magnetic resonance image (MRI) brain was performed on the sixth day of hospitalization, which demonstrated small areas of punctate acute infarcts involving the left cerebellar hemisphere, but no evidence of large vessel or brainstem strokes to suggest ongoing vertebrobasilar ischemia. The punctate infarcts are believed to be reflective of hypoperfusion or thromboembolic phenomena related to the initial injury to the left vertebral artery. The patient additionally underwent tracheostomy and percutaneous endoscopic gastrostomy tube placement on days 11 and 12 of admission, respectively. He was weaned from full ventilatory support and was tolerating tracheostomy collar prior to discharge. He was sent to a long-term acute care facility for rehabilitation on post-interventional procedure day 13. 

## Discussion

Acquired spinal AVFs are rare vascular malformations of the spine. Typically, they present after an iatrogenic injury during cervical spine surgery, central venous catheterization, chiropractic manipulations, diagnostic cerebral angiography, percutaneous nerve blocks, radiation therapy, or penetrating traumatic injury [1-5]. Spinal AVFs can present with symptoms of myelopathy or cervical neuralgia due to arterial blood reflux into spinal pial veins or after root compression by engorged epidural veins [6]. The timing of the development of symptoms is related to the flow velocity, venous drainage pattern, and lesion chronicity [7]. Arterial steal via rapid blood flow or altered venous drainage causing venous hypertension is the primary pathophysiologic mechanism by which acute neurologic deterioration occurs [8-9].

Blunt traumatic injury is an extremely rare etiology of spinal AVFs. We present the second reported case of cervical spinal AVF developing after blunt traumatic injury, sustained after a fall with fracture dislocations of the upper cervical spine. Heuer et al. reported the only other case in the literature of a spinal AVF developing after a blunt traumatic injury. Their report described a young male who presented after a motor vehicle accident with a C2 fracture. [10] The patient developed symptoms of upper and lower extremity weakness six hours after presenting with a normal neurologic exam. MRI of the cervical spine with magnetic resonance angiography and venography (MRA/MRV) demonstrated a probable high cervical cord AVF off of the right vertebral artery. [10] Digital subtraction angiography (DSA) showed complete transection of the distal cervical aspect of the right vertebral artery at the level of the base of C2 with anterograde and retrograde blood flow into a direct AVF, resulting in early filling of the right internal jugular vein and other external draining veins [10]. The patient was treated with coils proximal and distal to the vertebral artery transection [10]. Obliteration of the recipient venous pouch with NBCA and proximal sacrifice of the feeding vertebral artery as a method of shunt disconnection has not been previously described.

Historically, open surgery was used to treat spinal AVFs via proximal and distal trapping with surgical excision of the fistula [11-12]. However, there were significant morbidity and high failure rates via open surgery [11-12]. This was likely due to ineffective proximal vertebral artery ligation because of the extensive anastomotic circulation between the external carotid and vertebral arterial systems [13-14]. Due to advancements in endovascular technology and technique, spinal AVFs can be treated with low morbidity and high treatment success via obliteration of the fistula and preservation of flow in the parent vertebral artery [15-16]. Herrera et al. reported a case series of patients who developed spinal AVFs treated primarily via vertebral artery occlusion [7]. In their case series, patients presented with symptoms of cephalgia, cervical radiculopathy, subarachnoid hemorrhage, stiff neck, tinnitus, and myelopathy [7]. The authors demonstrated that endovascular treatment can be safely accomplished whenever the contralateral vertebral artery is adequate to supply both intracranial vertebral circulations, as was in our case.

## Conclusions

Cervical vertebral artery injury after blunt traumatic injury is extremely rare and vascular imaging in such instances is important, especially when an associated osseous injury is identified. In such cases of acquired vertebral artery AVF, endovascular treatment is a safe and effective method of shunt disconnection.

## References

[1] Cosgrove GR, Théron J (1987). Vertebral arteriovenous fistula following anterior cervical spine surgery. Report of two cases. J Neurosurg.

[2] Hayward R, Swanton H, Treasure T (1984). Acquired arteriovenous communication: complication of cannulation of internal jugular vein. Br Med J (Clin Ed.

[3] Inamasu J, Guiot BH (112). Iatrogenic vertebral artery injury. Acta Neurol Scand.

[4] Reizine D, Laouiti M, Guimaraens L (1985). Vertebral arteriovenous fistulas: clinical presentation, angiographical appearance and endovascular treatment—a review of twenty-five cases. Ann Radiol (Paris.

[5] Verrie`res D, Bernard C, Dacheux J (1986). Cervical arteriovenous fistulas after internal jugular catheterization. Ann Fr Anesth Reanim.

[6] De Bray JM, Bertrand P, Bertrand F (1986). Spontaneous arteriovenous fistulas of the vertebral artery: apropos of a case—review of the literature. Rev Med Interne.

[7] Herrera DA, Vargas SA, Dublin AB (2008). Endovascular treatment of traumatic injuries of the vertebral artery. AJNR Am J Neuroradiol.

[8] Aminoff MJ, Barnard RO, Logue V The pathophysiology of spinal vascular malformations. J Neurol Sci 23.

[9] Aminoff MJ, Logue V Clinical features of spinal vascular malformations. Brain.

[10] Heuer GG, Gabel BC, Bhowmick DA, Stiefel MF, Hurst RW, Schuster JM (2008). Symptomatic high-flow arteriovenous fistula after a C-2 fracture. Case report. J Neurosurg Spine.

[11] Aronson NI Traumatic arteriovenous fistula of the vertebral vessels. Angiographic demonstration and a rationale for treatment. Neurology l.

[12] Elkin DC, Harris MH Arteriovenous aneurysm of the vertebral vessels. Report of ten cases. Ann Surg.

[13] Bergquist E, Bergstrrm K, Hugosson R Complicated arteriovenous fistula after vertebral angiography. Neuroradiology.

[14] Dutton J, Isherwood I (1970). Iatrogenic vertebral arteriovenous fistulae. Minim Invasive Neurosurg.

[15] Albuquerque FC, Javedan SP, McDougall CG (2002). Endovascular management of penetrating vertebral artery injuries. J Trauma.

[16] Crowley RW, Medel R, Dumont AS (2009). Traumatic high flow vertebral-venous fistula presenting with delayed ischemic stroke: endovascular management with detachable coils and Amplatzer vascular plugs. Neurosurg Focus.

